# Effects of Microplastic Exposure against White Spot Syndrome Virus Infection in Pacific White Shrimp (*Penaeus vannamei*)

**DOI:** 10.4014/jmb.2402.02001

**Published:** 2024-03-22

**Authors:** Hye Jin Jeon, Sangsu Seo, Chorong Lee, Bumkeun Kim, Patharapol Piamsomboon, Ji Hyung Kim, Jee Eun Han

**Affiliations:** 1 Laboratory of Aquatic Biomedicine, College of Veterinary Medicine, Kyungpook National University, Daegu 41566, Republic of Korea; 2 Department of Veterinary Medicine, Faculty of Veterinary Science, Chulalongkorn University, Bangkok, Thailand; 3 Veterinary Medical Aquatic Animal Research Center of Excellence, Chulalongkorn University, Bangkok, Thailand; 4 Department of Food Science and Biotechnology, Gachon University, Seongnam 13120, Republic of Korea; 5 Institute for Veterinary Biomedical Science, Kyungpook National University, Daegu 41566, Republic of Korea

**Keywords:** Aquaculture, histopathology, microplastics, marine pollution, shrimp

## Abstract

Plastic waste has emerged as a major environmental concern in recent years. As plastic waste discharged into the marine environment, it undergoes a breakdown process, eventually accumulating in aquatic organisms in the form of microplastics (MPs). To date, reduced food intake, nutritional absorption, and impaired immune system are known adverse effects of MPs-exposed aquatic organisms. This study aims to investigate whether MP exposure accelerated white spot syndrome virus (WSSV) infection in Pacific white shrimp (*Penaeus vannamei*) via laboratory tests. Briefly, experimental shrimp were divided into four groups; WSSV (group 1); MP (group 2); WSSV + MP (group 3); and Control (group 4). No mortality was observed in group 2, group 4, and even in group 1. However, group 3 showed a cumulative mortality of 50% during the experimental period. The PCR assay results showed no WSSV in the other three groups (groups 1, 2, and 4), but the dead and alive shrimp collected from group 3 were confirmed to be infected with the virus. Histopathological examination revealed normal structures in the hepatopancreas, gill, and muscle tissues of group 4, whereas numerous abnormally shaped nuclei were detected in the gill tissue of group 2. Moreover, group 1 showed minor WSSV-related lesions with few basophilic inclusion bodies in the gills, interestingly, group 3 exhibited severe lesions with numerous basophilic inclusion bodies in the gills. In conclusion, this study confirmed the correlation between the viral disease of shrimp and MPs, which can cause significant economic losses to the shrimp aquaculture industry.

## Introduction

Crustaceans are among the most popular seafoods worldwide and contain beneficial nutrients for human health [1-3]. Shrimp are the most widely farmed crustaceans [4]. However, for several decades, shrimp farming has been threatened by viral diseases, which have caused significant economic losses worldwide [5-7]. White spot syndrome virus (WSSV) is one of the most severe viral pathogens affecting shrimp [8-10]. Its main symptom consists of distinct white spots (0.5–3.0 mm in diameter) under the shrimp’s carapace, and it has a high mortality of up to 100% within days [11]. Previous studies have confirmed that WSSV replication can be accelerated by various stress factors (*e.g.*, physical and chemical changes in water), increasing infection rates [12, 13].

Recently, there has been a noticeable surge in the utilization of plastic, and as a result, the amount of plastic waste has also increased. Such waste is released into the environment, where it breaks down due to various chemical and physical processes [14], resulting in particles of different sizes, *i.e.*, nanoplastics, microplastics (MPs), mesoplastics, and macroplastics [15]. Unfortunately, a considerable amount of plastic waste is discharged into the marine environment [16], after it undergoes multiple stages of degradation it is ingested and accumulated in marine organisms in the form of MPs [14, 17]. MP particles have a wide range of sizes (<5 mm) and a variety of shapes and colors due to differences in sources and production [15]. Once they have entered the food chain, MPs eventually bioaccumulate in species at higher trophic levels, such as mammalians [18, 19] or even humans [20]. As MPs degrade into smaller particles, they are more likely to be ingested by animals [17, 21, 22], resulting in a more severe impact [23, 24]. Moreover, MPs contribute significantly to environmental pollution, as they can adsorb pollutants such as antibiotics, heavy metals, and organic compounds in the water [25]. The adsorptive nature of MPs is a cause of even greater concern. The pollution of aquatic environments with plastic particles induces stress in aquatic organisms [26-28], which can disrupt their immune defense system [28, 29].

Numerous studies have investigated the tissue damage caused by MP accumulation in several aquatic organisms. For example, histopathological studies have examined the intestine of zebrafish [30], the brain tissue structure of crucian carp [31], the gills, hepatopancreas, and muscle of shrimp [32], and the liver and intestine of European sea bass [33] after exposure to MPs. In *Penaeus vannamei* (*P. vannamei*), known as Pacific white shrimp, histopathological changes in various organs, such as muscle, midgut gland, hepatopancreas, and gills, were observed after exposure to MPs at varying concentrations [32]. Recently, Shan *et al*. [34] have also reported a correlation between viral disease and MP exposure in shrimp; however, this correlation has not been yet confirmed based on histopathological examination. In this study, we hypothesized that MPs could accelerate viral disease in shrimp and tested this hypothesis by conducting a laboratory test. The experimental shrimp were infected with WSSV and then exposed to MPs. The correlation between the virus and MP exposure was then examined by determining mortality rates as well as by conducting a PCR assay and histopathological analysis.

## Materials and Methods

### Preparation for the Shrimp Test (Shrimp, WSSV Stock, and MPs)

Pacific white shrimp (*P. vannamei*) at the post-larval stage (approximately 0.6 g) were purchased from a local shrimp farm (Republic of Korea) and transported to the Laboratory of Aquatic Biomedicine, College of Veterinary Medicine, Kyungpook National University (Republic of Korea). The shrimp were reared to an average weight of 1.5 ± 0.05 g in 700-L maintenance tanks filled with aerated artificial seawater (25°C–28°C, 25 ppt salinity).

WSSV-tissue homogenates (WSSV stock, 1.62 × 10^8^ copies/μl), which had also been previously used in Han *et al*. [35], were quantified by qPCR analysis [9] and frozen at −80°C until use.

Fluorescent polystyrene microspheres (FSFR005, concentration: 1%) in a liquid state, purchased from Bangs Laboratories, Inc. (USA), were used as MPs in this study. The particles were colorless and had a mean diameter of 2.07 μm.

### Shrimp Test (WSSV and MP Exposure)

The experimental shrimp (average 1.5 ± 0.05 g, *N* = 32) were divided into four groups: group 1, WSSV; group 2, MP; group 3, WSSV + MP; and group 4, Control. The shrimp in each group (*N* = 8) were further divided into duplicates of four shrimp each and placed in different 12-L tanks (working water volume: 8 L). The artificial seawater in each tank was maintained as aforementioned rearing conditions. All tested shrimp were subjected to a 24-h fast before experimental infection.

The experimental shrimp (*N* = 8) of group 1 (WSSV) were exposed to WSSV once via a single feeding with the WSSV stock at 3% of the shrimp's average body weight on day 0. On day 2, 100 μl of 1× PBS was orally administered to each shrimp.

The experimental shrimp (*N* = 8) of group 2 (MP) were pre-exposed to the muscle of specific pathogen-free (SPF) shrimp once via a single feeding at 3% of the shrimp's average body weight on day 0. On day 2, 100 μl of MP solution was orally administered to each shrimp. MPs were previously diluted 20 times in 1× PBS (final concentration: 0.05%) and then mixed with yellow food dye (CHUN-WOO Co., Ltd, Republic of Korea) in a 1:1 v/v (yellow dye: MP) before administration.

The experimental shrimp (*N* = 8) of group 3 (WSSV + MP) were exposed to WSSV once via a single feeding with the WSSV stock at 3% of the shrimp's average body weight on day 0. On day 2, 100 μl of MP solution was orally administered to each shrimp.

The experimental shrimp (*N* = 8) of group 4 (Control) were exposed to the muscle of SPF shrimp once via a single feeding at 3% of the shrimp's average body weight on day 0. On day 2, 100 μl of 1× PBS was orally administered to each shrimp.

After exposure, the shrimp were fed three times a day with commercial shrimp feed containing 30% of crude protein at a total feed of 5% of their body weight for shrimp maintenance [36] and monitored every 12 h for 6 days. During the experiment, dead shrimp (on day 1 and day 3) and live shrimp (on day 6) were collected, and 30 mg of their gills were used for DNA extraction using the DNeasy Blood & Tissue Kit (Qiagen, Germany). The WSSV PCR assay was conducted as described in Nunan and Lightner [37].

### Histological Examination

At the end of the experiment (day 6), four moribund shrimp, one from each group, were fixed in Davidson’s AFA fixative (pH 3.0–4.0) [38] for 24 h and then transferred to 70% ethanol for histological examination. After dehydration in a graded ethanol series to absolute ethanol, the samples were embedded in paraffin and each tissue (hepatopancreas, gill, and muscle) was sectioned (4 μm thickness) following standard methods [39]. After staining with hematoxylin and eosin (H&E staining), the sections were analyzed by light microscopy. The severity of WSSV infection was determined on a scale from grade 1 (G1) to grade 4 (G4) [39, 40].

## Results

### Shrimp Test (WSSV and MP Exposure)

**Fig. 1:**
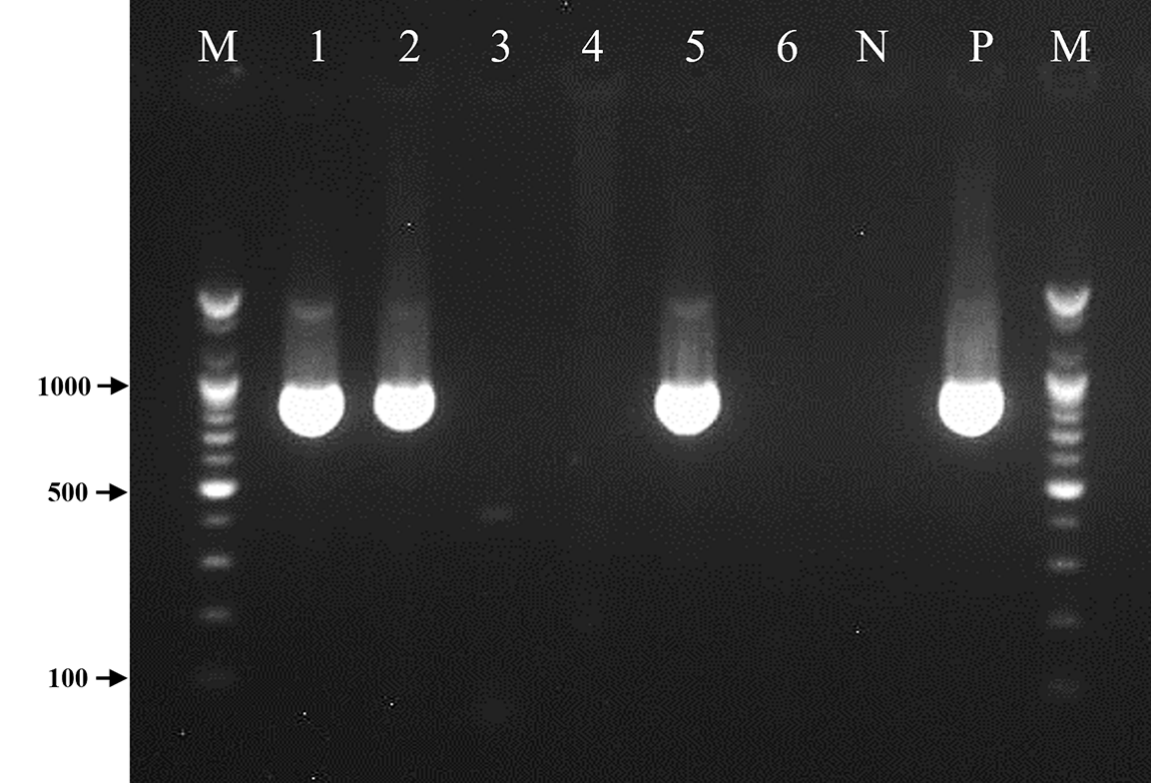
Result of WSSV PCR assay in dead shrimp during the experiment and live shrimp in each group on the last day (day 6). Lane M: 100-bp ladder; Lane 1: Dead shrimp in group 3 (WSSV+MP) within 24 h after MP exposure; Lane 2: Dead shrimp in group 3 (WSSV+MP) within 96 h after MP exposure; Lane 3: Live shrimp in group 1 (WSSV) on day 6; Lane 4: Live shrimp in group 2 (MP) on day 6; Lane 5: Live shrimp in group 3 (WSSV+MP) on day 6; Lane 6: Live shrimp in group 4 (Control) on day 6; Lane N: Negative control (DEPC-water); Lane P: WSSV positive control (941-bp).

During the experiment, no mortality was observed in group 1 (WSSV), group 2 (MP), and group 4 (Control) until the last day (day 6). However, in group 3, shrimp mortality was observed within 24 h, cumulatively reaching 50% during the entire experimental period (6 days). The PCR assay confirmed WSSV infection in the dead shrimp collected from group 3 (WSSV + MP). We also analyzed the live shrimp collected from each group on day 6, and interestingly, WSSV was detected in the individuals from group 3 (WSSV + MP) but not in those from group 1 (WSSV) (Table 1 and Fig. 1).

### Histological Examination

**Fig. 2:**
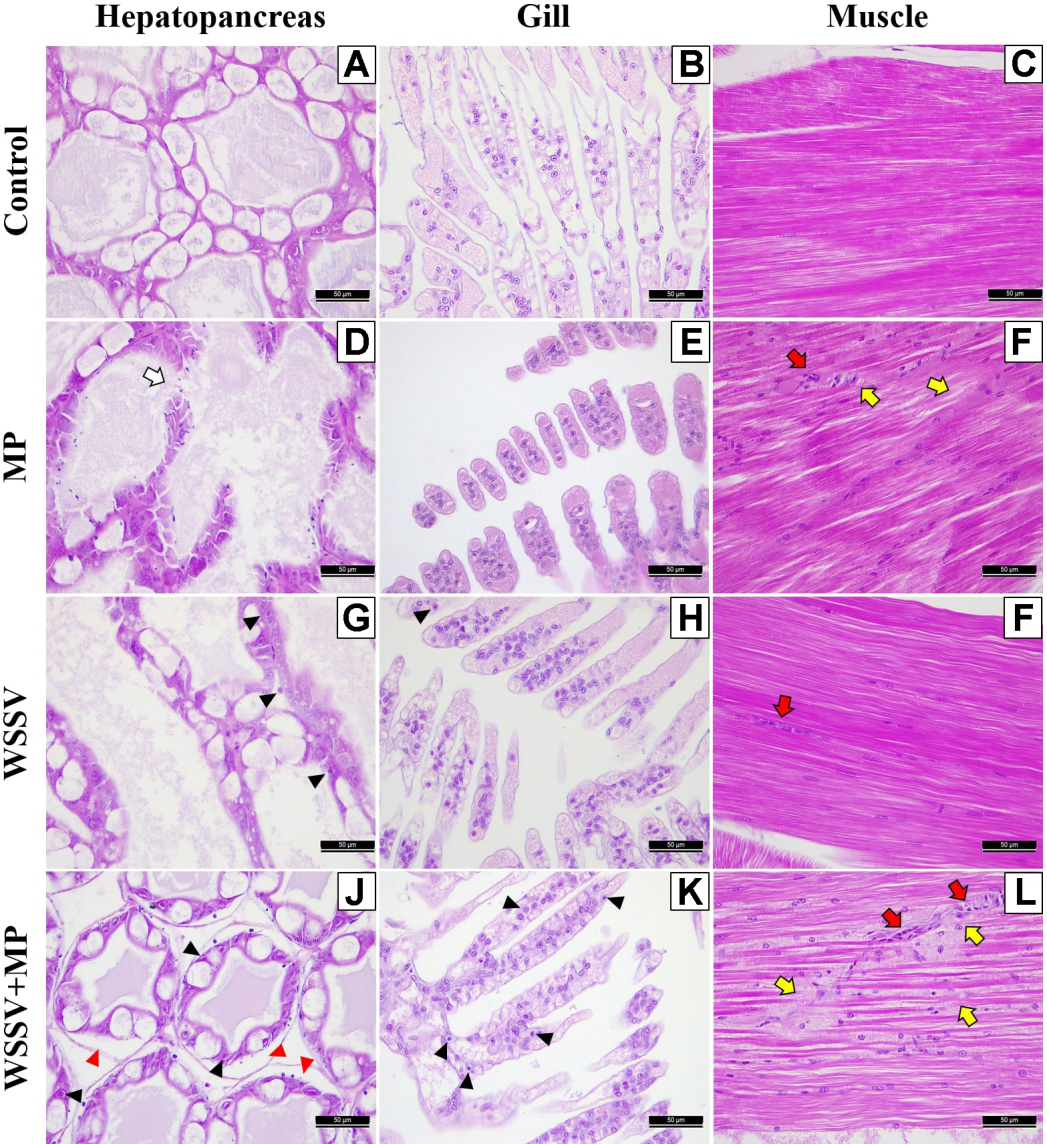
Photomicrographs of sections of shrimp tissues (hepatopancreas, gill, and muscle) for each experimental group. (**A**), (**B**), and (**C**): hepatopancreas, gill, and muscle of shrimp in group 4 (Control), respectively; (**D**), (**E**), and (**F**): hepatopancreas, gill, and muscle of shrimp in group 2 (MP); (**G**), (**H**), and (**I**): hepatopancreas, gill, and muscle of shrimp in group 1 (WSSV); (**J**), (**K**), and (**L**): hepatopancreas, gill, and muscle of shrimp in group 3 (WSSV+MP). All sections were stained with H&E. Scale bars = 50 μm.

We observed the histopathological effects of exposure to WSSV and MPs in the hepatopancreas, gill, and muscle tissues of the experimental shrimp. Normal structures of the hepatopancreas (Fig. 2A), gills (Fig. 2B), and muscle fibers (Fig. 2C) were observed in group 4 (Control). However, compared with the control, the individuals in group 2 (MP) exhibited collapsed tubular structures and B-cell losses (large vacuole in tubules; white arrow) in the hepatopancreas (Fig. 2D). Also, cytoplasmic effusion was observed in the gill tissue (Fig. 2E), and many nuclei showing hemocytic infiltration (red arrow) and slight lysis (yellow arrow) were observed in the muscle fibers (Fig. 2F).

The typical WSSV lesions were not observed in group 2 (MP) but were observed in group 1 (WSSV) and group 3 (WSSV + MP). In group 1 (WSSV), histopathological changes, including hypertrophied nuclei, were observed in the hepatopancreas (black arrowheads; Fig. 2G), and a few basophilic inclusion bodies in the gill tissue (black arrowheads; Fig. 2H), and along with few nuclei showing hemocytic infiltration (red arrow; Fig. 2I) were observed in the muscle fibers. However, in group 3 (WSSV + MP), hypertrophied nuclei (black arrowheads), and lumen separate from the basement membrane (red arrowheads) were observed in the hepatopancreas (Fig. 2J), and several basophilic inclusion bodies were observed in the gill tissue (black arrowheads; Fig. 2K). In addition, many nuclei showing hemocytic infiltration (red arrows), as well as infiltrated and dissolved muscle fibers (yellow arrows) were observed in the individuals of group 3 (Fig. 2L), but not in those of group 1 (WSSV). The histopathological examination revealed more severe WSSV lesions in group 3 (WSSV + MP) (G4) than in group 1 (WSSV) (G1).

## Discussion

Several studies have been conducted on the negative impact of plastics on various aquatic crustaceans, including crab, langoustine, and shrimp species. For example, MPs were shown to have negative effects on the growth of the Chinese mitten crab (*Eriocheir sinensis*) by inducing oxidative stress in the liver [41]. In the Norway lobster (*Nephrops norvegicus*), the feeding rate, body mass, and metabolic rate as well as the catabolism of stored lipids were shown to be reduced after exposure to MPs [42]. In Pacific white shrimp (*P. vannamei*), after exposure to nanoplastics, there were changes in nutritional compositions including a decrease in the levels of some essential amino acids and fatty acids, and alterations in intestine microbial activity were observed [43]. After exposure to MPs, the promotion of stress and immune responses was confirmed in *P. vannamei* [27]. In addition, the accumulation of plastic debris in shrimp was shown to make them more susceptible to various disease and stress factors [44], which could eventually lead to reduced production. However, the correlation between MPs and infectious viral diseases has been rarely studied in crustaceans.

White spot disease (WSD), which is caused by WSSV, is a notable viral infection primarily affecting farmed shrimp. When infected with this virus, shrimp develop white spots on the exoskeleton, which is unsightly, and the disease itself is highly infectious. WSD is recognized for its severity and is listed in the World Organization for Animal Health list of aquatic animal diseases [45, 46]. Moreover, WSD caused economic losses after the first outbreak in 1992, resulting in a production loss of over US$ 2 billion in China in three years [11]. Ecuador, the outbreak occurred in 1999 and resulted in losses of over US$ 1 billion from 1998 to 2001 [47], and over 100 million US$ in Panama, over 70 million US$ in Peru over 3 years [11]. The present study was conducted to evaluate whether MPs accelerate WSSV infection or mortality in juvenile *P. vannamei*.

Shan *et al*. [34] revealed that shrimp (larvae and adults) exposed to MP exhibited an increased susceptibility to WSSV and increased mortality; however, the study did not involve any histopathological examination. A previous study by Hsieh *et al*. [32], described histopathological examination of the shrimp tissues after MP exposure. However, shrimp were exposed to a small amount of MP using intramuscular injection, which is an unnatural exposure route for shrimp. The results of the histopathological examination conducted in the present study confirmed an accelerated WSSV infection in juvenile shrimp (1.5 ± 0.05 g) as well as increased mortality after MP exposure by oral administration. In line with Mohan *et al*. [48], the extent of tissue lesions is related to mortality data. In the present study, group 1 (WSSV) showed suspected WSSV-related lesions, but group 3 (WSSV + MP) showed serious WSSV-related lesions, and a higher mortality rate was observed in group 3 with severe WSSV-related lesions. The same WSSV stock with the same viral concentration (1.62 × 10^8^ copies/μl) was used to induce infection in both group 1 and group 3. However, group 1 did not show clinical symptoms or mortality, whereas group 3 exhibited a cumulative mortality of 50% by the 6th day after infection. This indicated that MP exposure amplified WSSV infection.

The results of this study highlighted the increased risk of infection that may result from exposure to MPs in shrimp and the consequent potentially elevated risk of significant economic losses in this aquaculture industry. The present study may contribute to research on the correlation between MP exposure and diseases in shrimp, as well as to raising awareness about protecting the marine environment. In addition, further studies will be necessary to obtain reliable data through long-term experiments based on the observation of histopathological changes due to MP exposure. Follow-up research is expected to confirm the correlation between MPs and other major diseases in shrimp farms as a potential factor for disease exacerbation.

## Figures and Tables

**Table 1:** Mortality rate (%), result of the white spot syndrome virus (WSSV) PCR assay, and observation of typical WSSV lesion by histological examination after exposure to WSSV and MPs.

Group	Mortality (%)	WSSV detection		
		PCR (dead)	PCR (live)	WSSV histology
Group 1 (WSSV)	0	NA^a^	-	+ (G1)^b^
Group 2 (MP)	0	NA	-	-
Group 3 (WSSV+MP)	50	+	+	+ (G4)
Group 4 (Control)	0	NA	-	-

^a^NA: not applicable. ^b^Classified to grades by histopathological lesion [38, 39].
